# Effects of continuous glucose monitoring in enhanced recovery after colorectal cancer patient surgery with type 2 diabetes (application of CGM in the ERAS process of CRC patient with diabetes)

**DOI:** 10.3389/fmed.2025.1464071

**Published:** 2025-06-03

**Authors:** Qinbo Wang, Yuan Zhou, Wanzhen Wang, Yingjuan Ou, Xia Wu, Junrong Chen, Xiaoyan Li, Hua Li

**Affiliations:** 1 Department of Pharmacy, The Sixth Affiliated Hospital, Sun Yat-Sen University, Guangzhou, China; 2 Department of Graceland Medical Center, The Sixth Affiliated Hospital, Sun Yat-Sen University, Guangzhou, China; 3 Biomedical Innovation Center, The Sixth Affiliated Hospital, Sun Yat-sen University, Guangzhou, China; 4 Department of Gastroenterology, The Sixth Affiliated Hospital, Sun Yat-Sen University, Guangzhou, China; 5 Department of General Practice, The Sixth Affiliated Hospital of Sun Yat-Sen University, Guangzhou, China

**Keywords:** continuous glucose monitoring, colorectal cancer, diabetes, surgery, ERAS

## Abstract

**Objectives:**

This study aimed to evaluate the impact of Continuous Glucose Monitoring (CGM) on enhanced recovery after surgery (ERAS) outcomes in colorectal cancer (CRC) patients with type 2 diabetes mellitus (T2DM).

**Methods:**

We conducted an observational cohort study of adult patients (≥18 years) with T2DM undergoing abdominal surgery for pathologically confirmed CRC. Exclusion criteria included history of other malignancies, preoperative infections, or incomplete clinical data. Participants were stratified into two groups: the exposed group (CGM monitoring) and the control group (conventional glucose analyzer [CGA]). Primary outcomes included glycemic variability, surgical complications, ERAS milestones, and patient satisfaction scores.

**Results:**

Among 181 enrolled patients (CGM = 81, CGA = 100), CGM demonstrated superior glycemic control compared to CGA, with significantly lower mean daily glucose levels at postoperative day 1 (9.52 ± 2.53 vs. 10.37 ± 2.26 mmol/L, *p* < 0.05) and day 3 (9.36 ± 1.82 vs. 10.64 ± 1.84 mmol/L, *p* < 0.05). The CGM group showed better clinical outcomes including improved anastomotic healing (*p* < 0.05), shorter time to first flatus (*p* < 0.05), and reduced length of hospitalization (*p* < 0.05). Patient satisfaction scores were significantly higher in the CGM group (32.42 ± 3.33 vs. 29.81 ± 2.98, *p* < 0.05).

**Conclusion:**

CGM provides superior perioperative glucose monitoring in diabetic CRC patients, particularly during the critical 72-h postoperative period. The technology facilitates early detection of acute hyperglycemia, promotes wound healing, and accelerates recovery within ERAS protocols. These findings support the clinical value of CGM implementation in surgical management of T2DM patients with CRC.

## Introduction

Colorectal cancer is a prevalent malignancy of the digestive system, accounting for approximately 10% of global cancer diagnoses and related deaths each year (1, 2). Surgery plays a critical role in treatment, and perioperative blood glucose levels are an important factor. The extensive scope of radical resection and prolonged surgical stimulation in patients with colorectal cancer can lead to insulin insensitivity, stress hyperglycemia, increased risk of postoperative complications, hindered wound healing, and increased risk of wound infection (3). Studies have indicated that diabetic patients undergoing surgical treatment have more than three times the incidence and mortality rate of postoperative complications compared to non-diabetic patients (4). Furthermore, preoperative chemotherapy, psychological factors, surgery, and other variables can increase the risk of hyperglycemia in patients with colorectal cancer and diabetes (5). Therefore, it is crucial to explore continuous and comprehensive methods for the management of blood glucose during the perioperative period to develop personalized blood glucose control programs that enhance the patient recovery after surgery.

Continuous Glucose Monitoring (CGM) systems are increasingly being utilized as alternative methods for monitoring blood glucose levels in patients with diabetes receiving insulin therapy (6, 7). This allows for personalized diet plans and improved quality of life by reducing the risk of high or low blood sugar fluctuations (8, 9). The CGM sensor measures interstitial fluid glucose levels every minute and automatically stores data every 15 min (10). Combined CGM therapy involves continuous drug infusion along with blood sugar monitoring, which allows dynamic adjustment of insulin doses based on changes in blood sugar levels (11). While some studies have shown benefits for outpatients with T1D and T2D (12–14), there is clinical evidence for the use of CGM in the ERAS process of colorectal cancer patients with T2D (15, 16). This study aimed to investigate the benefits of continuous glucose monitoring during surgery in colorectal cancer patients with diabetes while exploring how CGM can be integrated into perioperative medication management to optimize blood glucose control services within enhanced recovery after surgery (ERAS) program.

## Patients and methods

Patient data were retrieved from the database of the Sixth Affiliated Hospital of Sun Yat-sen University from September 2020 to December 2024, including individuals with colorectal cancer (CRC) and type 2 diabetes mellitus (T2DM) who underwent resection surgery. The inclusion criteria were as follows: (I) endoscopic biopsy or postoperative pathological diagnosis of CRC, (II) age ≥18 years, presence of type 2 diabetes, (III) underwent resection surgery, and (IV) fasting plasma glucose levels measured before the initiation of first-line therapy and during follow-up treatment. The exclusion criteria were a history of other malignancies, preoperative infection, and difficulty in obtaining relevant data. The enrolled patients had previously received treatment with diet alone, oral monotherapy, or a combination of oral agents including glucagon-like peptide 1 agonists or insulin therapy. Blood glucose levels, surgical complications, and rehabilitation outcomes were assessed. Patients who received continuous glucose monitoring were classified into the exposed group, whereas those who used conventional glucose analyzers were classified into the control group. Continuous glucose monitoring sensor data after hospital discharge were downloaded using Libre View software and exported to standard Excel data files for further statistical analysis by the statistician. Each point-of-care blood glucose measurement was paired with the corresponding continuous glucose monitoring value within 5 min and used for accuracy analysis as a reference for the treatment protocol. This study was approved by the Ethics Committee of the Sixth Affiliated Hospital of the Sun Yat-sen University. Written informed consent was obtained from all patients.

### Continuous glucose monitoring (CGM)

**Figure 1:**
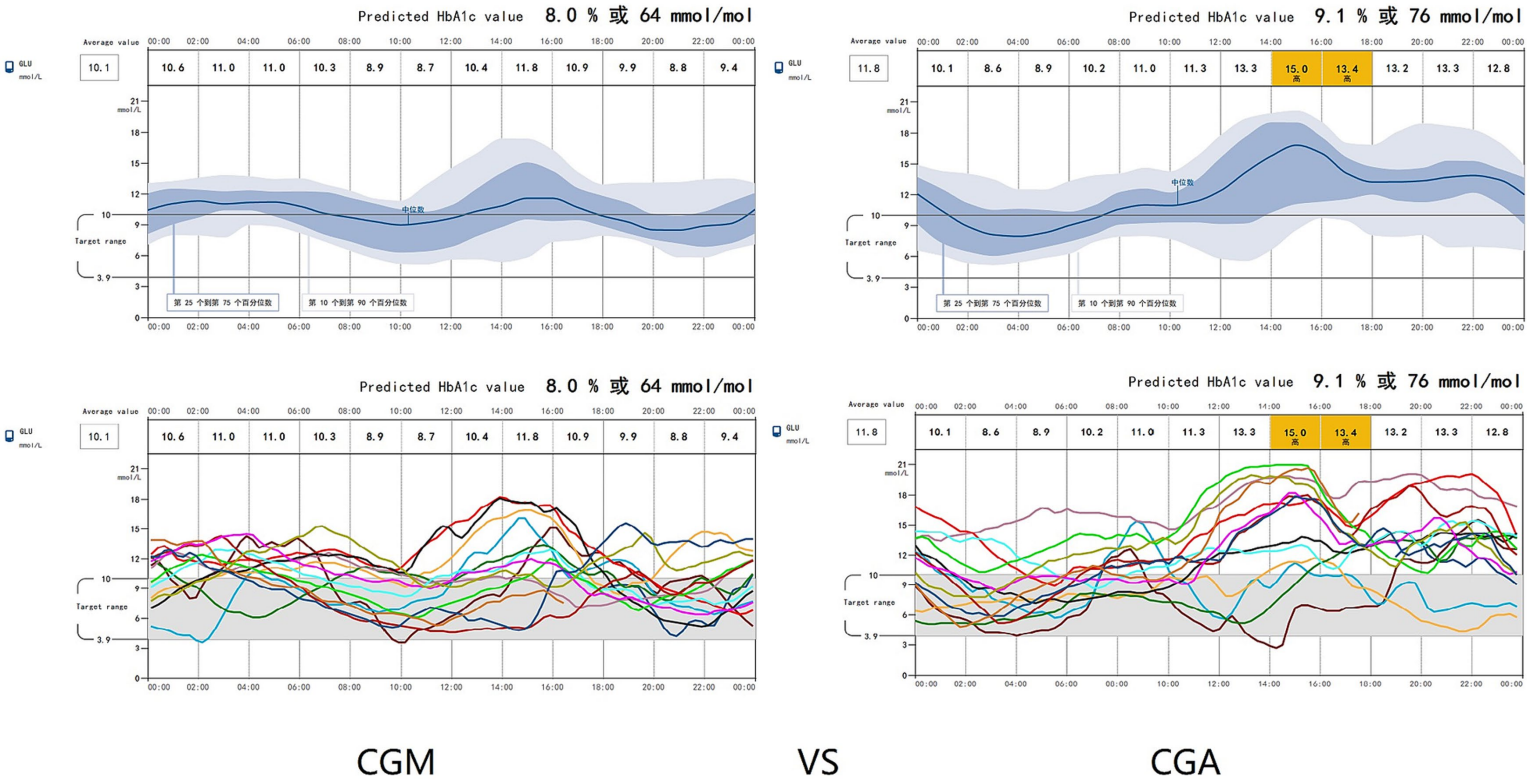
Blood glucose waveform over 0–14 days monitored by CGM. The blood glucose levels monitored by Continuous Glucose Monitoring (CGM) are depicted in the waveform, with red indicating when the patient’s blood glucose falls below the target control range and orange representing levels above the target range. **(A)** Illustrates the waveform of a patient with stable blood glucose fluctuations. The daily blood glucose range remained relatively consistent, with sharply fluctuations observed on the 4th, 7th, and 8th days after-surgery. **(B)** Displays the waveform of a patient with significant blood glucose variability. Hypoglycemia (indicated by the red region) was detected on the second day, but the patient’s blood glucose levels promptly returned to normal following intervention. In the subsequent days, the patient experienced pronounced upward fluctuations in blood glucose levels.

**Figure 2:**
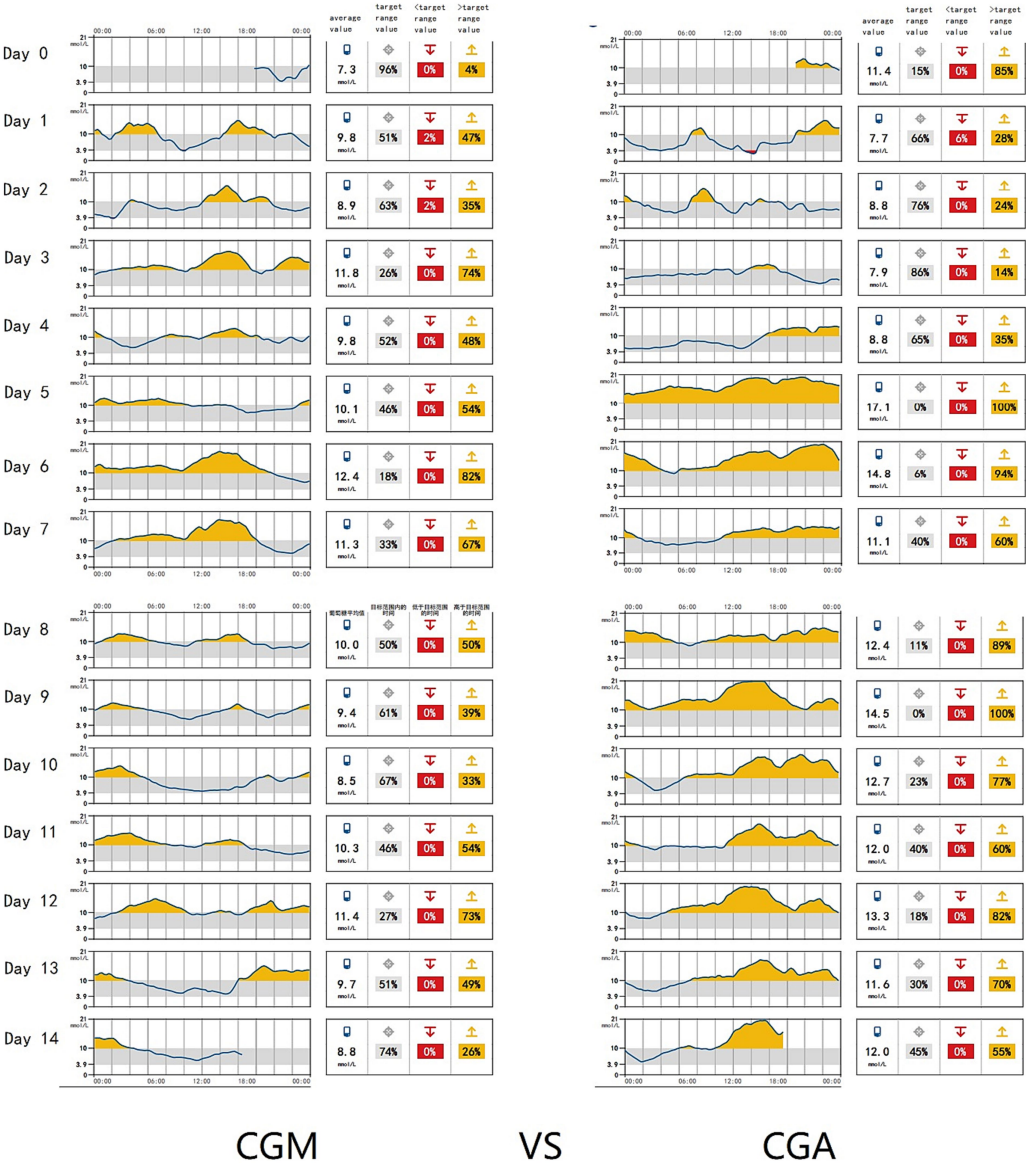
24-hour blood glucose waveform of CRC Patients monitored by CGM and predicted HbA1c values. Continuous Glucose Monitoring (CGM) was used to track the 24-h blood glucose patterns of colorectal cancer (CRC) patients, and the corresponding HbA1c values were predicted based on the data. **(A)** Displays the blood glucose waveform of a patient with stable glucose fluctuations. The average daily blood glucose level was 10.1 mmol/L, and the predicted HbA1c value was 8.0%. **(B)** Illustrates the blood glucose waveform of a patient with significant glucose variability. The average daily blood glucose level was 11.81 mmol/L, with a peak glucose level reaching 15.0 mmol/L, the predicted HbA1c value for this patient was 9.1%.

Patients in the CGM group received a continuous infusion of recombinant human insulin connected to a Medtronic iPro2 CGM device. The skin around the navel was sterilized with 75% ethanol up to a distance of 5 cm from the navel. A probe was inserted into the needle aid at an angle of 45°. After removing the needle core, it was connected to the monitor, which recorded an average value every 5 min based on the signals received every 10 s (shown in Figures 1, 2). The blood glucose levels monitored by CGM are depicted in the waveform, with red indicating when the patient’s blood glucose falls below the target control range and orange representing levels above the target range. Figure 1A Illustrates the waveform of a patient with stable blood glucose fluctuations. The daily blood glucose range remained relatively consistent, with sharply fluctuations observed on the 4th, 7th, and 8th days after surgery. Figure 1B displays the waveform of a patient with significant blood glucose variability, the patient’s blood glucose levels promptly returned to normal following intervention. Patients had access to their own reader device or could use an app on their smartphone to scan the sensor for real-time glucose readings. Insulin dosage adjustments were immediately made by the doctors according to the blood glucose graph. Blood glucose levels at 8:00, 14:00, and 22:00 were measured using finger glucometers and compared with instantaneous glucometer readings to correct sequential rod glucometers. Human insulin injection was utilized, the insulin was manufactured by Novo Nordisk (China Pharmaceutical Co., Ltd.). The dosage for each patient was carefully adjusted based on the data obtained from continuous glucose monitoring (CGM), with each dose adjustment not exceeding 4 units to ensure precise and safe glycemic control.

### Conventional glucose analyzer (CGA)

Patients in the control group underwent insulin treatment according to the national guidelines and bedside CGA capillary glucose testing every 6 h (at 6:00 AM, 10:00 AM, 2:00 PM, 6:00 PM, and 10:00 PM). Before pricking their ring finger with a disposable fingertip blood glucose test needle in its thin part near the abdominal area of the finger, the nurses disinfected it using a cotton ball soaked in 75% ethanol. For patients with hypoglycemia or hyperglycemia, the blood glucose level was measured, and the insulin dosage was adjusted according to the blood glucose value.

### Measurements

Data collected from the database included patient demographics, blood glucose fluctuations, surgical complications, ERAS rehabilitation, and patient satisfaction. Our primary objective was to compare glycemic control in hospital settings between the CGA and CGM groups, including mean daily glucose levels. The blood glucose levels were measured from admission to discharge. The second endpoint of the study was the incidence of postoperative complications such as wound infection, urinary tract infection, pneumonia, poor healing of the anastomosis, ketoacidosis, blood glucose fluctuation, thrombus, ileus, or abdominal distension. Patient satisfaction was assessed using the Diabetes Treatment Satisfaction Questionnaire Status version (DTSQs) before discharge.

### Statistical analysis

SPSS software (version 25.0, SPSS Inc., Chicago, IL, USA) was used for data analysis. Normally distributed data were represented as mean ± standard deviation, the Kruskal–Wallis test was used to compare continuous variables. The data were evaluated with a 95% confidence interval and 5% significance level. Statistical significance was set at *p* < 0.05.

## Results

### Clinical demographics

A total of 181 colorectal cancer patients with diabetes were enrolled in the study, with 81 patients undergoing continuous glucose monitoring (CGM) and 100 patients undergoing conventional glucose monitoring (CGA). The mean ages of Group CGM and Group CGA were 61.22 ± 11.1 and 57.64 ± 12.3 years, respectively. The mean admission HbA1c levels were 6.21 and 6.38% (mmol/mol), while the mean BMI values were 22.04 ± 0.86 and 20.07 ± 2.33 kg/m^2^, and the mean duration of diabetes was found to be 10.72 ± 4.48 and 9.78 ± 5.12 years for Group CGM and Group CGA, respectively. Both before and after propensity score matching, the two groups demonstrated comparable baseline characteristics with no statistically significant differences in age distribution, duration of diabetes history, HbA1c level, medication, comorbid conditions, or surgical site distribution (*p* > 0 0.05; shown in Table 1).

**Table 1:** Characteristics of patient in CGA and CGM groups.

Patient characteristics	Unmatched			Matched		
	CGM (*n* = 81)	CGA (*n* = 100)	*p*-value	CGM (*n* = 72)	CGA (*n* = 83)	*p*-value
Age	61.22 ± 11.10	57.64 ± 12.3	0.245	60.48 ± 14.38	57.04 ± 11.67	0.116
Diabetes history	10.72 ± 4.48	9.78 ± 5.12	0.545	11.68 ± 6.24	11.99 ± 5.14	0.801
BMI	22.04 ± 0.86	20.07 ± 2.33	0.865	21.46 ± 1.46	20.87 ± 1.82	0.303
HbA1c (%)	6.21 ± 1.45	6.38 ± 1.13	0.105	6.75 ± 1.71	6.15 ± 0.97	0.055
Male Gender	46 (56.79%)	60 (60.0%)	0.215	33 (45.83%)	52 (62.65%)	0.302
Medication	72 (88.89%)	94 (94.00%)	0.210	68 (94.44%)	80 (96.39%)	0.281
Comorbid conditions	57 (70.37%)	56 (56.00%)	0.089	51 (70.83%)	52 (62.65%)	0.062
Surgical site distribution (Colon and Rectal)	80 (94.30%)	100 (100.00%)	0.351	70 (97.22)	100 (100.00%)	0.465

### Blood glucose fluctuation

There were no significant differences between the two groups in terms of HbA1C before and after surgery. The mean daily glucose during the hospital stay was significantly higher by CGA blood glucose compared with CGM (10.37 ± 2.26 vs. 9.52 ± 2.53 mg/dL) (*p* < 0.05) the 1st day after surgery (shown in Table 2), and with mean blood glucose of 10.64 ± 1.84 mg/dL in the CGA group compared to 9.36 ± 1.82 mg/dL in the CGM group at the 3th day after surgery. There were no significant differences between the two groups in terms of blood glucose the 5th day after surgery.

**Table 2:** The comparison of blood glucose and HbA1C.

Timeline	Glucose index	CGM (*n* = 81)	CGA (*n* = 100)	*t* value	*p*-value
Preoperative	FBG	6.12 ± 0.54	6.81 ± 0.88	1.0121	0.109
	2hPG	7.82 ± 1.71	8.48 ± 1.60	0.512	0.562
	HbA1C (%)	6.04 ± 0.84	6.38 ± 1.13	0.925	0.182
1 d after surgery	2hPG	9.52 ± 2.53	10.37 ± 2.26	−2.12	0.023
3 d after surgery	2hPG	9.36 ± 1.82	10.64 ± 1.84	−1.53	0.021
5 d after surgery	2hPG	9.24 ± 1.88	9.36 ± 2.10	0.167	0.553
7 d after surgery	2hPG	9.65 ± 2.05	9.56 ± 2.65	0.213	0.632
Before discharge	FBG	6.07 ± 0.32	6.12 ± 0.76	−1.32	0.201
	2hPG	9.19 ± 2.62	9.33 ± 2.73	−1.49	0.545
	HbA1C (%)	7.08 ± 1.89	6.94 ± 2.31	0.432	0.212

FBG, fasting blood glucose; 2hPG, 2-h post-meal blood glucose.

### Complications and hypoglycemia detection

Postoperative complications were objectively compared between the two groups. Postoperative complications included infection, poor anastomotic healing, ketoacidosis, stomal leak, thrombus, ileus, and abdominal distension. There were significant differences between the CGM and CGA groups in terms of anastomotic healing (*t* = −1.891, *p* < 0.05, Table 3). These results indicate that the CGM method is more effective in improving anastomotic healing. All complications resolved after appropriate treatment measures were taken. Furthermore, we determined the number of hypoglycemia episodes lasting at least 15 min with a reading above 12.0 mmol/L detected by CGM. The incidence of hyperglycemia in the CGM group was significantly lower than that in the CGA group (*t* = −2.164, *p* < 0.05). There were no episodes of severe hypoglycemia with a reading below 3.9 mmol/L detected by either method. The proportion of patients with hypoglycemia was similar, with no significant difference.

**Table 3:** Comparison of postoperative complications between the two groups.

Complications	CGM (*n* = 81)	CGA (*n* = 100)	*t* value	*p*-value
Infection	6 (7.41%)	6 (6.00%)	0.000	0.821
Poor anastomosis healing	2 (2.47%)	4 (4.00%)	−1.891	0.021
ketoacidosis	0 (0.00%)	1 (1.00%)	−1.000	0.125
Stomal leak	1 (1.23%)	4 (4.00%)	−1.763	0.081
Thrombus	0 (0.00%)	1 (1.00%)	−1.000	0.223
Ileus	4 (4.94%)	3 (3.00%)	0.000	1.237
Abdominal distension	0 (0.00%)	1 (1.00%)	−1.023	0.223
Hyperglycemia	18 (22.22%)	32 (32.00%)	−2.164	0.001
Hypoglycemia	7 (8.64%)	4 (4.00%)	1.001	0.520
Complications rate	5.21%	6.22%	−2.42	1.241

### Eras rehabilitations

We observed that the CGM group had a shorter hospital stay than the CGA group did. There were significantly differences between two groups in terms of first exhaust time, and hospital stays, among which the first exhaust time was 22.52 ± 0.53 h in the CGM group compared to 18.98 ± 0.73 h in the CGA group (*p* < 0.05), hospital stays was 6.59 ± 2.63 days in the CGM group compared to 8.86 ± 2.47 days in the CGA group (*p* < 0.001; shown in Table 4).

**Table 4:** The comparison of rehabilitation index.

Index	CGM (*n* = 81)	CGA (*n* = 100)	*t* value	*p*-value
First exhaust time (h)	22.52 ± 0.53	18.98 ± 0.73	2.821	0.001
Catheter indwelling time (h)	35.45 ± 2.67	34.90 ± 2.17	1.203	0.305
Time of the first ambulation session (h)	13.75 ± 0.42	15.86 ± 0.81	−1.266	0.412
Postoperative hospital stays (d)	6.59 ± 2.63	8.86 ± 2.47	−5.89	0.000

### Questionnaire results

A total of 181 questionnaires were sent for feedback collection purposes and received back responses from 126 participants. DTSQ scores were obtained for 62 and 64 participants in the CGM and CGA groups, respectively. According to self-reports provided by participants, there were significant differences in terms of treatment satisfaction rates (5.92 ± 0.38 vs. 5.51 ± 0.71), convenience (5.76 ± 0.54 vs. 4.46 ± 1.47), flexibility (5.52 ± 0.38 vs. 4.59 ± 1.16), and recommended treatment plan (5.65 ± 0.87 vs. 4.79 ± 1.21). CGM group showed better feedback than CGA group with the score of 32.42 ± 3.33 in the CGM group compared to 29.81 ± 2.98 in the CGA group (*p* < 0.05, shown in Table 5).

**Table 5:** The comparison of satisfaction between two groups.

DTSQ score	CGM (*n* = 81)	CGA (*n* = 100)	*t* value	*p*-value
Q1 current treatment	5.92 ± 0.38	5.51 ± 0.71	2.859	0.026
Q4 convenience	5.76 ± 0.54	4.46 ± 1.47	4.588	0.000
Q5 flexibility	5.52 ± 0.38	4.59 ± 1.16	5.382	0.043
Q6 understanding treatment	5.45 ± 0.72	5.19 ± 1.01	2.622	0.667
Q7 recommend treatment plan	5.65 ± 0.87	4.79 ± 1.21	4.735	0.001
Q8 continue treatment plan	5.02 ± 0.44	5.30 ± 0.79	0.105	0.981
Total score	32.42 ± 3.33	29.81 ± 2.98	8.506	0.001
Q2 frequent of hyperglycemia	0.85 ± 0.54	0.72 ± 0.62	0.645	0.461
Q3 frequency of hypoglycemia	1.20 ± 0.52	1.25 ± 0.68	−0.242	0.382

A total of 8 items, including treatment satisfaction, self-perceived hyperglycemia, self-perceived hypoglycemia three dimensions. Treatment satisfaction includes 6 items (current treatment, convenience, flexibility, understanding treatment, recommend treatment plan, continue treatment plan), from 0 very dissatisfied to 6 very satisfied, with a total score of 36; Self-perceived hyperglycemia and self-perceived hypoglycemia ranged from less than 0 to 6 most of the time, and the higher the score, the more frequently patients reported feeling.

## Discussion

The primary objective of perioperative blood glucose management is ensuring patient safety and stability. Our findings demonstrated that continuous glucose monitoring (CGM) is significantly more effective in promoting anastomosis healing (*p* < 0.05). Elevated blood glucose levels have been linked to delayed wound healing and increased infection rates, whereas recurrent and severe hypoglycemia may negatively affect patient outcomes, leading to higher morbidity, mortality, prolonged hospital stay, readmission, and increased medical costs (17, 18). A meta-analysis involving surgical patients with diabetes revealed that maintaining perioperative blood glucose levels was associated with reduced perioperative mortality and stroke (19–21). In this observational cohort study of hospitalized colorectal diabetes patients receiving insulin treatment, we compared the effects of continuous glucose monitoring (CGM) and conventional Glucometer Analysis (CGA). Our results indicated a trend toward lower post-surgery glucose concentrations with CGM measurements, the CGM group exhibited lower blood glucose levels compared to the CGA group, and the difference was statistically significant (*p* < 0.05). This suggests that the use of CGM monitoring may not inherently lead to superior glycemic control, nor does it directly correlate with accelerated patient recovery or earlier discharge. Instead, the clinical value of CGM in this context appears to lie primarily in its predictive capabilities and real-time glucose monitoring. By providing healthcare professionals with objective, data-driven insights, CGM facilitates more informed and timely clinical decision-making, thereby enhancing the precision of subsequent interventions. Using established tools for evaluating CGM performance (22–24), we observed that CGM detected more episodes of nocturnal and persistent hyperglycemia attributed to parenteral nutritional support during gastrointestinal surgery than limited daily testing using CGA. Including insulin in parenteral mixed solutions presents challenges in effectively controlling insulin infusion rates owing to drug interactions or precipitation, resulting in significant fluctuations in blood sugar levels and an increased risk of hypoglycemia. Colorectal cancer patients are predominantly middle-aged and elderly individuals (61.22 ± 11.10 vs. 57.64 ± 12 years old), with reduced metabolic function making them more susceptible to insulin resistance and impaired insulin secretion. Patients with diabetes are also prone to perioperative blood sugar fluctuations; therefore, serum glucose levels should be monitored along with HbA1c testing, which is crucial for evaluating treatment effectiveness in diabetes management (25). However, HbA1c is not suitable for monitoring acute changes in the perioperative blood glucose levels.

The mean daily glucose during the hospital stay was significantly higher by CGA blood glucose compared with CGM (10.37 ± 2.26 vs. 9.52 ± 2.53 mg/dL) (*p* < 0.05) at the 1st day after surgery, and with mean blood glucose of 10.64 ± 1.84 mg/dL in the CGM group compared to 9.36 ± 1.82 mg/dL in the CGM group at the 3th day after surgery, indicating that continuous blood glucose monitoring can serve as a reliable reference for clinicians to evaluate patients’ blood glucose values and facilitate timely treatment of abnormalities. According to American Diabetes Association Professional Practice Committee, once insulin therapy is started, a target glucose range of 7.8–10.0 mmol/L is recommended for the majority of critically ill and noncritically ill patients. Although 1 mmol/L reduction was observed in our research, it’s still has clinical significant (26). A comprehensive 24-h blood glucose profile can identify nocturnal or asymptomatic hypoglycemia (27, 28). The key to perioperative management is to establish a clear target blood glucose level and monitor it frequently so that the treatment regimen can be adjusted accordingly (29). Dynamic glucometers can rapidly provide real-time glucose readings and access historical data in order to generate visual graphs (30, 31). The use of dynamic glucometers compensates for the limitations associated with traditional monitoring methods by objectively and accurately reflecting patients’ blood sugar levels at different time intervals, thereby aiding more effective overall control of their blood sugar status (32). In our study, CGM detected more hyperglycemic events, detecting up to 8.64%% of asymptomatic hypoglycemic events occurring in the evening or early morning between dinner and 06:00 h. These findings are clinically significant, as they highlight the potential impact of CGM on improving the detection of blood glucose fluctuations in hospitalized patients (33), which may lead to better regulation of insulin therapy and reduce morbidity and mortality associated with hypoglycemic events (32, 34–36).

Postoperative complication analysis demonstrated comparable risk profiles between CGM and CGA monitoring, with both modalities showing favorable postoperative outcomes. This may be attributed to the shorter hospital stay for rapid rehabilitation, and absence of adverse reactions. Following resection for low rectal cancer, a permanent stoma on the abdominal wall is required to replace the physiological function of the anus. However, a high blood sugar status can affect wound healing and increase the likelihood of incision infection. A previous study found that a longer duration of diabetes was an independent risk factor for perioperative hypoglycemia. Patients with a diabetes duration of ≥ 10 years had a 2.736 times increased risk of hypoglycemia compared to those with a diabetes duration of <10 years (37, 38), but there were no significant differences in patient diabetes history in our study. Significant differences were observed in first exhaust time and hospital stay durations between the two groups; average first exhaust time was 22.52 ± 0.53 h in the CGM group shorter than 18.98 ± 0.73 h in the CGA group, while hospital stay was 6.59 ± 2.63 days shorter than 8.86 ± 2.47 days in the CGM group. Once intestinal function was restored, the frequency of hyperglycemia after transitioning from parenteral nutrition to enteral nutrition was lower in the CGM group than that in the CGA group. The dynamic glucose monitoring system can provide dietary guidance based on patients’ intake and exercise recommendations according to their blood glucose fluctuations, facilitating rapid postsurgical recovery while ensuring that controlled insulin dosages are administered after meals to prevent significant blood sugar fluctuations that may hinder wound healing and early mobilization. The efficacy of diabetes treatment should not be evaluated solely by HbA1c levels, as they should also focus on patient-reported outcomes (PROs) such as patient satisfaction, wellbeing, and quality of life (39). The questionnaire survey showed that the patients were satisfied with the CGM method, with a score of 32.42 ± 3.33 compared to 29.81 ± 2.98 in the CGA group, which may be related to reduced daily invasive procedures with sustained blood glucose outcomes.

## Conclusion

Continuous glucose monitoring (CGM) provides clinicians with comprehensive insights into perioperative glycemic dynamics in diabetic patients, particularly during the critical 72-h postoperative window that significantly impacts ERAS outcomes. These findings underscore the importance of structured glucose monitoring protocols and targeted patient education. Special attention should be given to high-risk populations, including patients with cachexia-inducing tumors or long-standing diabetes (>10 years duration), who require intensified monitoring to mitigate hypoglycemic risks.

Our results demonstrate that real-time detection of acute hyperglycemia through CGM facilitates optimized glycemic control, thereby promoting wound healing and accelerating postoperative recovery. The implementation of early enteral nutrition following intestinal function recovery further enhances metabolic management by enabling personalized dietary and activity guidance. This multimodal approach not only improves glycemic stability but also enhances patient self-management capacity, quality of life, and treatment satisfaction.

Nevertheless, CGM have some limitations. Several important limitations warrant consideration: Clinical Implementation Barriers: The current adoption of perioperative CGM remains limited by cost considerations. Future cost-effectiveness analyses should stratify patients by surgical complexity and diabetes severity to identify subgroups deriving maximal benefit. Technological Constraints: All available CGM systems measure interstitial glucose with an inherent 5–15 min physiological lag relative to blood glucose, potentially reducing accuracy during rapid glycemic fluctuations. Additionally, current devices require twice-daily capillary glucose calibration. Measurement Limitations: While the DTSQ effectively evaluates satisfaction with diabetes technologies (e.g., insulin pumps, CGM), its psychometric properties remain unvalidated in surgical populations, and potential nonresponse bias merits consideration when interpreting satisfaction metrics.

This study’s non-randomized design introduces possible selection and confounding biases. Future research should prioritize randomized controlled trials to validate these findings, with particular attention to high-risk subgroups such as insulin-dependent patients or those with long diabetes duration. Such investigations could further clarify the optimal role of CGM in surgical diabetes management. We acknowledge that the unlicensed, unverified Chinese DTSQ translation was used (40, 41).

## Data Availability

The original contributions presented in the study are included in the article/supplementary material, further inquiries can be directed to the corresponding authors.
