# Correction: Effects of continuous glucose monitoring in enhanced recovery after colorectal cancer patient surgery with type 2 diabetes (application of CGM in the ERAS process of CRC patient with diabetes)

**DOI:** 10.3389/fmed.2026.1797153

**Published:** 2026-02-26

**Authors:** Qinbo Wang, Yuan Zhou, Wanzhen Wang, Yingjuan Ou, Xia Wu, Junrong Chen, Xiaoyan Li, Hua Li

**Affiliations:** 1 Department of Pharmacy, The Sixth Affiliated Hospital, Sun Yat-Sen University, Guangzhou, China; 2 Department of Graceland Medical Center, The Sixth Affiliated Hospital, Sun Yat-Sen University, Guangzhou, China; 3 Biomedical Innovation Center, The Sixth Affiliated Hospital, Sun Yat-Sen University, Guangzhou, China; 4 Department of Gastroenterology, The Sixth Affiliated Hospital, Sun Yat-Sen University, Guangzhou, China; 5 Department of General Practice, The Sixth Affiliated Hospital of Sun Yat-Sen University, Guangzhou, China

**Keywords:** continuous glucose monitoring, colorectal cancer, diabetes, surgery, ERAS

The references [40, 41] were not cited in the article.

40. Bradley C. The diabetes treatment satisfaction questionnaire: DTSQ. In: Bradley C, editor. *Handbook of Psychology and Diabetes: A Guide to Psychological Measurement in Diabetes Research and Practice*. Abingdon: Routledge (1994). p. 111–32.

41. Bradley C, Lewis KS. Measures of psychological well-being and treatment satisfaction developed from the responses of people with tablet-treated diabetes. *Diabet Med*. (1990) 7:445–51. doi: 10.1111/j.1464-5491.1990.tb01421.x

These citations have now been inserted in the Conclusion and should read: “We acknowledge that the unlicensed, unverified Chinese DTSQ translation was used [40, 41].”

The original version of this article has been updated.

